# Development of a therapeutic vaccine targeting Merkel cell polyomavirus capsid protein VP1 against Merkel cell carcinoma

**DOI:** 10.1038/s41541-021-00382-9

**Published:** 2021-10-05

**Authors:** Dan Xu, Sheng Jiang, Yue He, Xiang Jin, Gan Zhao, Bin Wang

**Affiliations:** 1 Key Laboratory of Medical Molecular Virology (MOE/NHC/CAMS), School of Basic Medical Sciences, Fudan University, Shanghai, China; 2 Shanghai Zerun Biotech Co., LTD, Shanghai, China; 3 Present Address: Advaccine Biopharmaceutics (Suzhou) Co. LTD, Suzhou, China

**Keywords:** Adjuvants, Protein vaccines

## Abstract

Merkel cell carcinoma (MCC) is a rare but aggressive skin cancer with a high mortality rate, while Merkel cell polyomavirus (MCV) has been pointed as the causative agent of MCC. A better prognosis of MCC associated with a high level of antibodies against the capsid protein VP1 suggests that anti-VP1 immune response might be essential against MCC growth. In the current study, we developed a VP1-target vaccine formulated with CRA. Using a tumorigenic CMS5-VP1 tumor model, the vaccine-induced a potent antitumor efficacy in a dose-dependent manner was evidently demonstrated and mainly mediated by both VP1-specific CD4^+ ^and CD8^+ ^T-cell responses against the growth of CMS5-VP1 tumors in vaccinated BALB/c mice since the depletion of CD4^+ ^and CD8^+ ^T cells reverse the antitumor effects. Thus, immunotherapy with this vaccine represents a novel approach for the clinical treatment of aggressive MCV-related MCC in humans.

## Introduction

Merkel cell carcinoma (MCC) is a rare but aggressive skin cancer with a higher mortality rate than observers with melanoma, aging or immunosuppressed individuals are at increased risk of MCC^1^. Merkel cell polyomavirus (MCV) is the causative agent, which has been identified in 43–100% of MCC tumors^1,2^. Serological studies have indicated that MCV infection mainly occurs during early childhood, and the prevalence of MCV in the health population increases with age. This virus represents part of the skin microbiota in a latent, non-replicative state after infection^3–5^.

MCV is the first human polyomavirus to mediate tumorigenesis^6^. MCV is a naked double-stranded DNA virus with the circular genome of ~5.4 kbp encompassed the early gene encoding oncoprotein T antigen (TA) and the late gene encoding capsid protein VP1, VP2 and VP3. The TA could be alternatively spliced into LT (the large TA), sT(small TA), and 57 kT, all of which share a 78-amino acid N terminus^7^. Despite widespread MCV, MCC is rare due to a very low probability of viral genomic integration followed by C-terminal truncation of TA to render the viral genome incapable of replication but promote cell cycle progression and immunosuppression or loss specific surveillance for MCV epitopes^8^. The capsid proteins VP1, VP2, and VP3 are expressed after the onset of viral DNA replication to self-assemble into viral particles of ~55 nm diameter with icosahedral symmetry, VP1 is the major capsid protein to forming the viral particle and define the binding site required for infection, with the minor capsid protein VP2 may facilitate a post-attachment stage of MCV infectious, the role of VP3 in MCV infectious is still unclear^9,10^.

Various therapies have been used for MCC clinic treatments, but the outcome of clinical prognosis is poor, with a low rate of 5-year overall survival and high risk of recurrence owing to immune compromise^2,11^. CD8^+ ^T cells have been reported to strongly influence overall survival and disease-specific survival in MCC. Moreover, MCV might be a prognostic factor to prompt a host immune response involving CD4^+ ^and CD8^+ ^T cells^8^.

Despite the success of vaccines against human papillomaviruses (HPV) and HBV, there was no reported prophylactic vaccine against MCV yet. Owing to a small population of MCV-infected individuals develop MCC, a prophylactic vaccine for MCV is likely not cost-effective. By contrast, a therapeutic vaccine target MCV may be an effective therapy for MCV-related MCC. There are several strategies to develop therapeutic vaccines. Appropriate tumor-specific targets are crucial for cancer immunotherapy, and viral antigens are the preferred target for virus-induced cancers. Like another oncovirus, TA is the oncoprotein, and truncation of TA is critical for MCC development. Indeed, two DNA vaccines targeting TA have been reported with antitumor effect through increasing antigen-specific CD4^+^ or CD8^+^ T cells in tumor-bearing mice^12,13^. The fact is that MCC patients had high-titer antibodies against TA in comparison with low titer in healthy individuals, in contrast to lower antibodies against TA associated with poor prognosis and high risk of recurrence, high levels of VP1 antibodies result in increased overall survival and lower probability of recurrence^14^. Furthermore, the cellular responses could be induced by VP1 as well as TA^15^. It is considered that a therapeutic vaccine target MCV capsid protein VP1 may improve the antitumor effects.

Unlike preventive vaccines inducing a humoral immune response, the therapeutic vaccine is thought to enhance cellular responses by stimulating antigen-specific CD8^+^ T cells. Since MCC patients are aging or immunosuppressed, energizing ineffectively primed T cells is particularly important. Even more, existing CD8^+^ T cells have likely encountered cognate tumor antigen due to the significant antigen burden of cancer, and as a result, they exhibit decreased effector function and a state similar to exhaustion^16,17^.

Adjuvant selection is as important as appropriate antigen. Cytokines or TLR agonists have been reported as potential adjuvants with antitumor effects. A recombinant HBV vaccine adjuvanted with GM-CSF and IFN-*α* resulted in the clearance of HBeAg and HBsAg of HBV-infected mice^18^. Some TLR agonists have been reported with potential adjuvant effects in preclinical studies^19–21^.

In the current study, several VP1-targeting vaccine candidates were developed with full-length VP1 and various adjuvant compositions. Of these candidates, a vaccine comprised of VP1/CRA could generate VP1-specific cellular immunity and facilitate the eradication of CMS5-VP1 tumors in a murine model. This study demonstrates that a combination of adjuvants with recombinant capsid protein VP1 of MCV could effectively induce anti-VP1 responses and lead to the eradication of VP1-expressed tumors.

## Results

### MCV capsid protein VP1 expression and purification

A codon-optimized VP1 was synthesized and cloned into a pET28a plasmid and then expressed by using an *E.coli* protein expression system (Supplementary Fig. 1a). The final protein product, herein named VP1, is approximated 50 kDa in size on sodium dodecyl-sulfate polyacrylamide gel electrophoresis (SDS-PAGE) and carries a His-tag to facilitate purification as detected by a Rabbit anti-VP1 antibody by Western Blot (Supplementary Fig. 1b). To generate an antibody against VP1, 10 µg VP1 adjuvanted with 500 µg Al(OH)_3_ was intramuscularly injected into naïve BALB/c mice twice by a 2-weeks interval, then sera from immunized mice were collected two weeks after the last vaccination. The sera would be used as an identification antibody for VP1 expression in the CMS5-VP1 cell line.

### Establishment of MCV VP1 murine tumor model

CMS5 cells (a murine sarcoma cell line) were transduced with pcDH-VP1 containing an optimized gene encoding VP1 under the control of a CMV promoter to generate tumorigenic VP1-expressing cell line, CMS5-VP1. A single clone of CMS5-VP1 cells was analyzed to identify VP1 expression using a flow cytometer with the gating strategy shown in the Supplementary Fig. 2a. CMS5-VP1 cells specifically expressed the VP1 compared with CMS5 cells (Supplementary Fig. 2b). Furthermore, the level of VP1 expression was identified by Western blot analysis (Supplementary Fig. 2c). A tumorigenicity study of CMS5-VP1 was performed as naïve BALB/c mice were inoculated with 1 × 10^6^ of CMS5-VP1 or CMS5 cells subcutaneously to observe tumor growth (Supplementary Fig. 2d). VP1-expressing in CMS5-VP1 and CMS5 tumor model were identified by Western blot (Supplementary Fig. 2e), cell lysate from CMS5-VP1 tumors (lane 2) demonstrated a specific VP1 band, and the band was absent in cell lysate from CMS5 tumor (lane 1). Thus, a murine VP1-expressing tumorigenic cell line CMS5-VP1 was generated successfully.

### Evaluation of adjuvant effects on the VP1 therapeutic vaccine

**Fig. 1:**
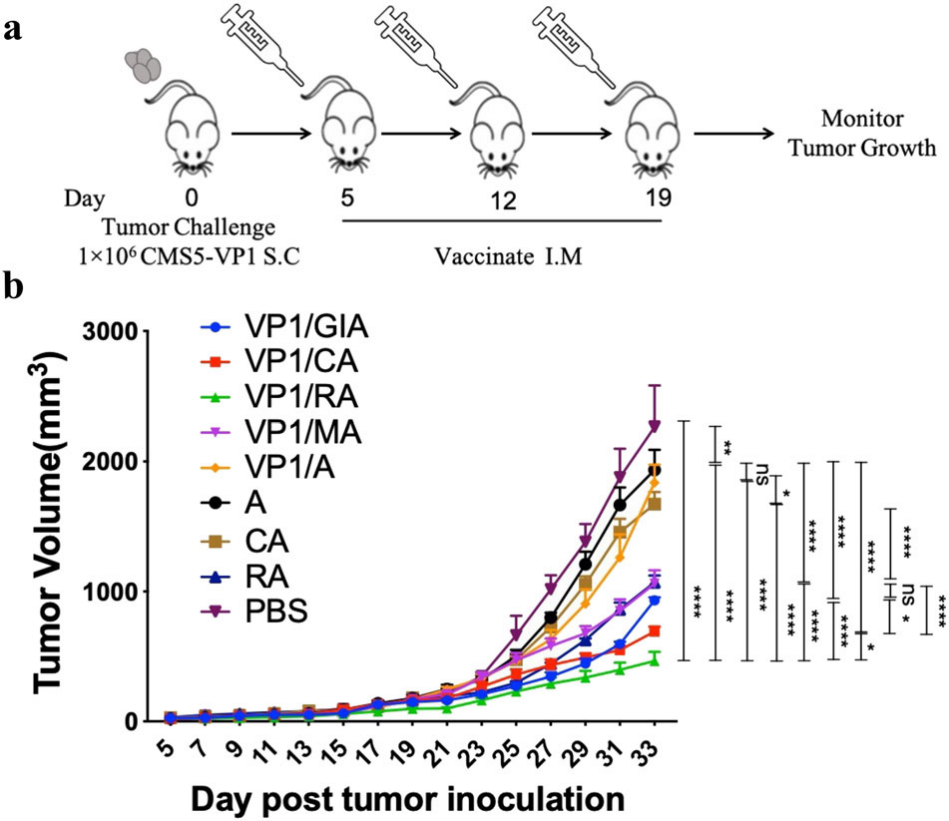
Antitumor effects of vaccine candidates. MCV VP1 murine tumor model was created by subcutaneously (S.C.) inoculation 1 × 10^6^ of VP1-expressing tumor cells (CMS5-VP1) into naïve BALB/c mice (5 per group). **a** Vaccine candidates or controls formulated as described in Materials and Methods were given to the tumor-bearing mice on days 5, 12, and 19 post tumor inoculation. **b** Tumor growth was measured every 2 days with digital calipers after vaccination and euthanized when tumor volumes reached 2000 mm^3^ or when tumors began to impair mobility or ulcerate. Statistics by ordinary two-way ANOVA, *p*:0.1234(NS), 0.0332(*), 0.0021(**), 0.0002(***), <0.0001(****).

Vaccine candidates VP1/GIA, VP1/CA, VP1/RA, VP1/MA, and VP1/A were formulated as mentioned in Material and Methods. CMS5-VP1 tumor-bearing mice were immunized thrice with 1-week intervals starting from day five post tumor inoculation (Fig. 1a). These candidates, especially VP1/CA and VP1/RA, could significantly inhibit CMS5-VP1 growth compared to control groups (Fig. 1b) as VP1 adjuvanted with CA or RA could generate strong antitumor effects (VP1/CA vs. VP1/A *p* < 0.0001, VP1/RA vs_._ VP1/A *p* < 0.0001), this indicated that CA and RA could serve as potential therapeutic adjuvants to facilitate antitumor immune effects.

### The combination of CpG and R848 improves antitumor effects

**Fig. 2:**
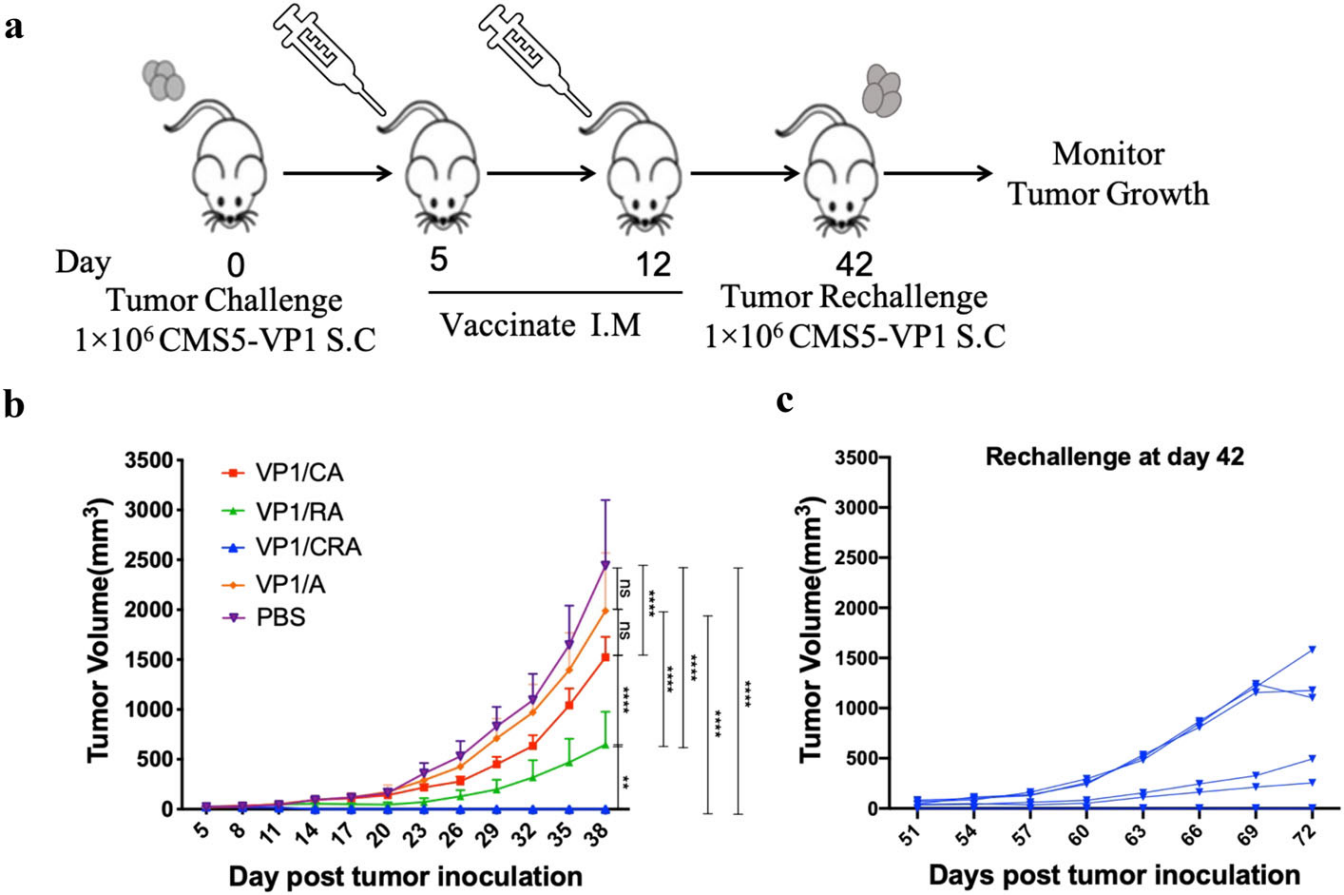
Twice immunization of VP1/CRA induced strong but not durable antitumor effect. **a** Schematic represents the treatment schedule. Naive BALB/c mice were subcutaneously inoculated with 1 × 10^6^ of CMS5-VP1 tumor cells in the left flank back on day 0. When tumors were palpable, the tumor-bearing mice were divided randomly and followed by twice intramuscular administration of VP1/CRA, VP1/CA, VP1/RA, VP1/A or PBS in the left hind limp on days 5 and 12. **b** Tumor volume was measured with digital calipers every 3 days. Mice were euthanized when tumor volumes reached 2000 mm^3^ or when tumors began to impair mobility or ulcerate. **c** After tumor completely regressed in group of VP1/CRA, tumor-free mice were rechallenged with 1 × 10^6^ of CMS5-VP1 on day 42. Tumor growth of rechallenged CMS5-VP1 were measured with digital calipers every 3 days and tumor volumes were calculated. Statistics by ordinary two-way ANOVA, *p*:0.1234(NS), 0.0332(*), 0.0021(**), 0.0002(***), <0.0001(****).

Having demonstrated that both CA and RA as adjuvant could facilitate antitumor effects. We hypothesized that combining CRA might further enhance antitumor efficacy. VP1/CRA vaccine was formulated as mentioned previously, and antitumor effects were investigated, as shown in Fig. 2a. Tumor-bearing mice were immunized with VP1/CRA twice by intramuscular administration with a 1-week interval. It revealed that the treatment either with VP1/CA or VP1/RA resulted in partial inhibition of CMS5-VP1 tumor growth while VP1/A had no effects. In contrast, the VP1/CRA immunized group shown complete rejection of CMS5-VP1 tumors (Fig. 2b). To confirm the enhanced antitumor results generated by the VP1/CRA vaccine, tumor-free mice were used to rechallenge with the same number of CMS5-VP1 cells again in the right flank back on day 42. Although VP1/CRA could induce antitumor effects to inhibit CMS5-VP1 tumors in the first place, but not sufficient to inhibit the growth of these rechallenged CMS5-VP1 tumor cells (Fig. 2c). Only 1 of 6 mice was successfully overcome the rechallenge, and 2 of them were developed with a smaller tumor burden. These results prompted that additional treatments might be necessary to induce an even stronger and more durable antitumor effect.

**Fig. 3:**
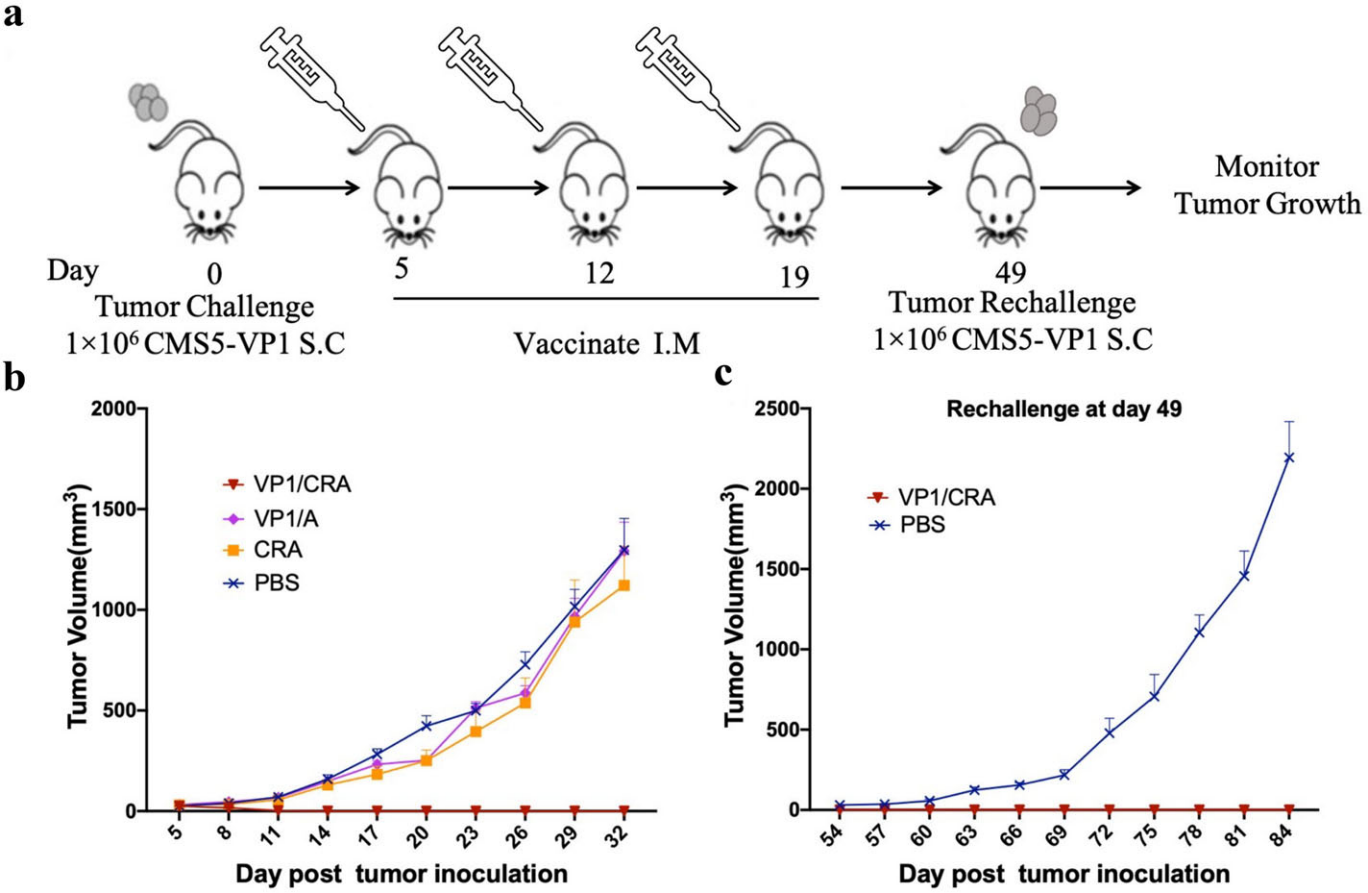
Triple immunization of VP1/CRA induced strong and durable antitumor effect. **a** An outline of the treatment schedule. CMS5-VP1 tumor cells at 1 × 10^6^ per mouse were subcutaneously inoculated into the left flank back of BALB/c mice on day 0. **b** When tumors were palpable on day 5, tumor-bearing mice were randomly divided and immunized with VP1/CRA, VP1/A, CRA, and PBS thrice at 1-week intervals. **c** To evaluated the durable antitumor effects induced by triple treatment of VP1/CRA, tumor-free mice were rechallenged with CMS5-VP1 tumor cells at 1 × 10^6^ per mouse in the right flank back on day 49. Tumors were measured with digital calipers and tumor volumes were calculated.

To test this notion, the triple-treatment was performed as depicted in Fig. 3a. Tumor-bearing mice were immunized with VP1/CRA thrice with 1-week intervals, regression of CMS5-VP1 tumor growth as shown in Fig. 3b, consistent with previous results. It was exhilarated that triple-treatment of VP1/CRA not only cause CMS5-VP1 regression for the first challenge and the rechallenged CMS5-VP1 cells (Fig. 3c). These results demonstrated that the antitumor response induced by VP1/CRA is in a dose-dependent manner.

### Induction of multi-functional T-cell responses

**Fig. 4:**
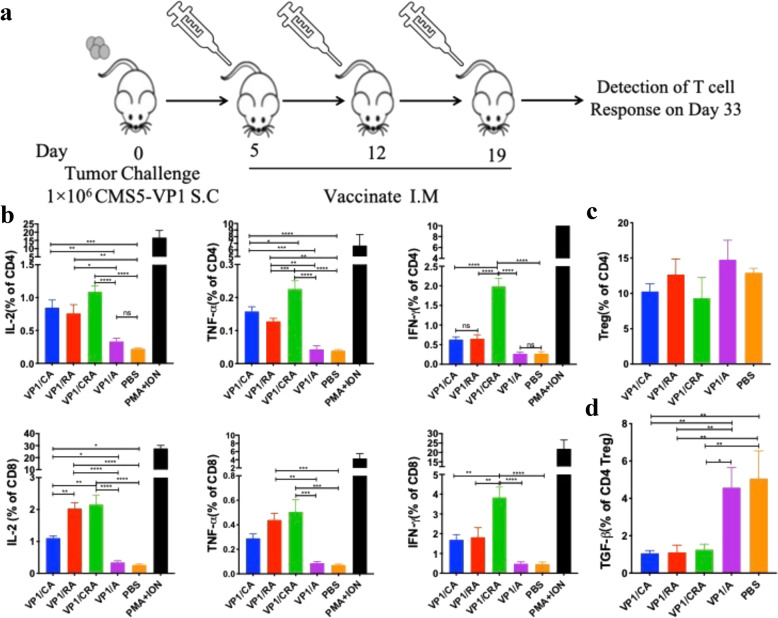
Treatment of VP1/CRA resulted in activation of effective T cells in CMS5-VP1 tumor-bearing mice. **a** Treatment schedule was depicted. Naive BALB/c mice were subcutaneously inoculated with CMS5-VP1 tumor cells (1 × 10^6^ per mice) in the left flank back on day 0. When tumors were palpable, tumor-bearing mice were randomly divided into five per group and immunized thrice starting from day 5 with 1-week intervals. **b** Percentage of IL-2, TNF-*α*, and IFN-*γ* expressing CD4^+ ^or CD8^+ ^T cells in Figure 3A were statistically analyzed with ordinary one-way ANOVA. **c** Percentage of CD4^+ ^Tregs cells in Figure 3B. **d** Percentage of TGF-β Tregs in Figure 3B. *P:* 0.1234(NS), 0.0332(*), 0.0021(**), 0.0002(***), <0.0001(****).

To assess whether T-cell response is the primary driver of antitumor effects elicited by VP1/CRA triple-treatment regimens (Fig. 4a). VP1-specific T-cell responses were analyzed by flow cytometer.

Both VP1 and PMA/Iono stimulated splenocytes were analyzed for cytokine expression by using a flow cytometer with the gating strategy shown in the Supplementary Fig. 3a. Cytokines of IL-2, TNF-*α*, and IFN-*γ* expressed in CD4^+ ^or CD8^+ ^T cells were presented in the Supplementary Fig. 3b. The statistics result illustrated that immunized with VP1/CRA could significantly enhance the expression of cytokines (Fig. 4b). Moreover, with the gating strategy shown in the Supplementary Fig. 3c, Treg cells (Tregs) in lymph node were analyzed (Supplementary Fig. 3d, upper panel), the statistical result of FOXP3 expression cells showed that there were no significant differences among groups of VP1/CRA, VP1/CA, VP1/RA, VP1/A or PBS (Fig. 4c). As transforming growth factor beta1 (TGF-β1) is a potent immunosuppression factor expressed by Tregs, TGF-β1-expressing Tregs were detected in the current study (Supplementary Fig. 3d, lower panel), and the statistical result has demonstrated that immunization of VP1/CA, VP1/RA or VP1/CRA resulted in a significant reduction of the percentage of TGF-β-expressing Tregs (Fig. 4d). This result suggests that VP1/CRA vaccine can activate T cells and may break the immune tolerance.

Apart from T-cell responses, to assess whether anti-VP1 antibody response would also contribute to antitumor effects simultaneously, serum samples were collected when mice were sacrificed. Antibody levels against VP1 were performed by enzyme-linked immunosorbent assay (ELISA) and exhibited high antibody levels against VP1 in sera from vaccinated mice without significant differences among vaccine VP1/CA, VP1/RA, VP1/CRA, and VP1/A (Supplementary Fig. 3e).

### A critical role of T cell for antitumor responses

**Fig. 5:**
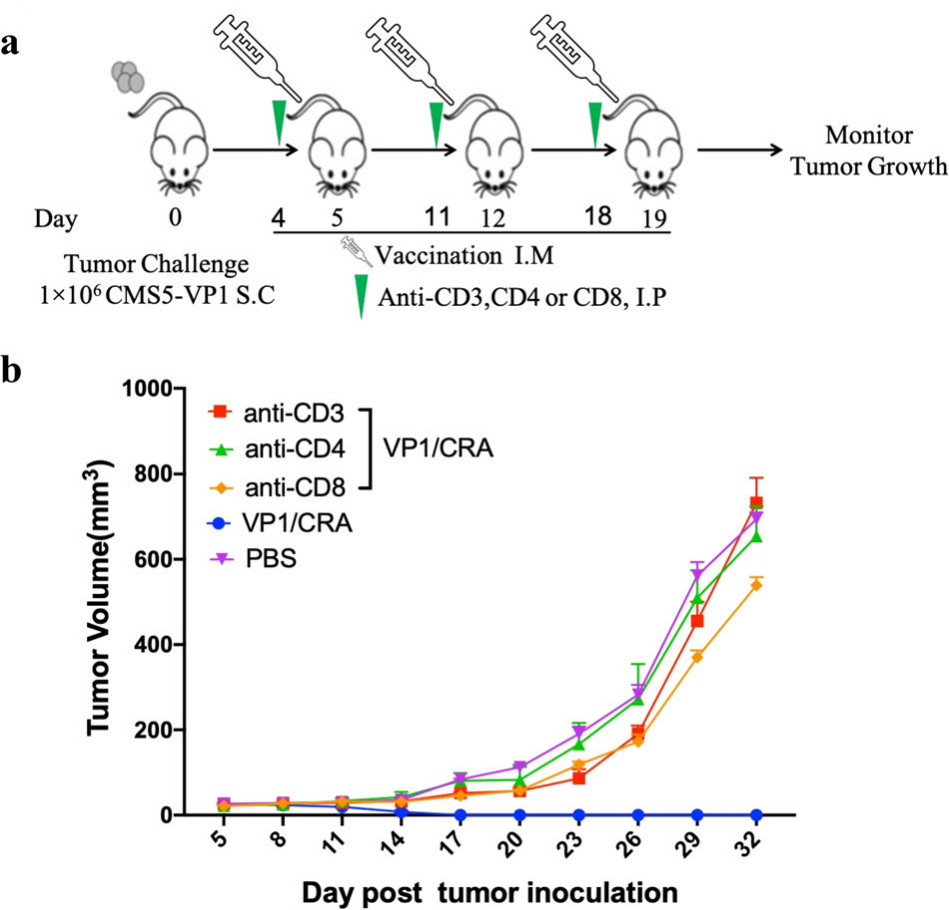
T cells were the main driver of the antitumor effect induced by VP1/CRA. The treatment schedule was depicted. Briefly, CMS5-VP1 tumor cells at 1 × 10^6^ per mouse were inoculated into naïve BALB/c mice subcutaneously in the left flank black on day 0. When tumors were palpable, tumor-bearing mice were randomly divided into five mice per group, tumor-bearing mice were intraperitoneally injected with anti-CD3, anti-CD4, or anti-CD8 monoclonal antibodies once a week starting from day 4, followed by the immunization of VP1/CRA in the left hind limp once a week. **b** Tumors were measured with digital calipers every 3 days and tumor volume was calculated.

To demonstrate whether T cells were involved in the antitumor responses induced by the vaccine, CMS5-VP1 tumor-bearing mice were immunized with VP1/CRA following depletion of CD3^+ ^, CD4^+ ^or CD8^+ ^T cells by administrating anti-CD3, anti-CD4, or anti-CD8 monoclonal antibodies at the day before immunization, respectively (Fig. 5a). Tumor volume was monitored and recorded, which is shown in Fig. 5b. It was consistent with previous results that tumors became regression after VP1/CRA treatments. Such antitumor response was dramatically abolished in the absence of CD3^+ ^, CD4^+ ^, or CD8^+ ^T cells, demonstrating the essential role of T-cell responses in antitumor effects induced by the vaccine.

### The antigen-specific antitumor effect induced by VP1/CRA vaccine

**Fig. 6:**
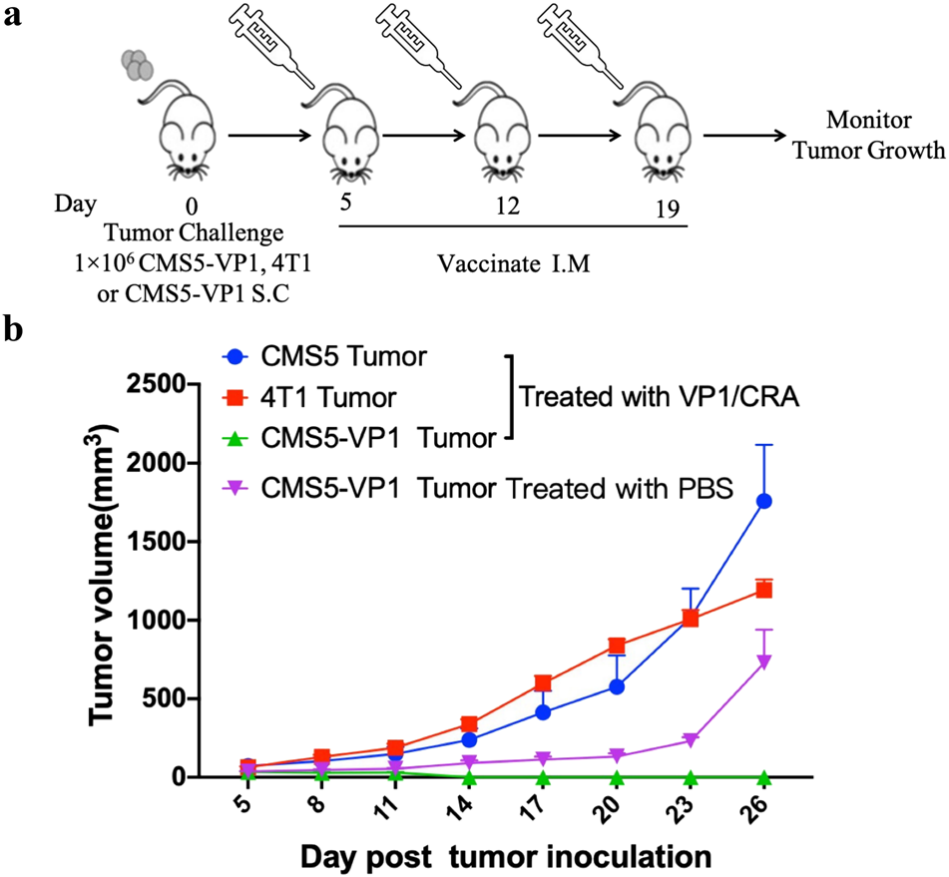
VP1/CRA vaccine could induce the antigen-specific antitumor effect. **a** Treatment schedule was depicted. Naive BALB/c mice were subcutaneously inoculated with CMS5-VP1, CMS5, or 4T1 tumor cells at 1 × 10^6^ per mouse with five mice per group on day 0. When tumors were palpable, tumor-bearing mice were vaccinated in the left hind limp thrice with 1-week intervals starting from day 5. **b** Tumors were measured with digital calipers every 3 days and tumor volumes were calculated. Mice were euthanized when tumor volumes reached 2000 mm^3^ or when tumors began to impair mobility or ulcerate.

To assess whether the antitumor effect is VP1-specific, CMS5-VP1 tumor-bearing mice and non-VP1-expressing tumor-bearing mice from either CMS5 or 4T1 were immunized with VP1/CRA thrice with 1-week intervals (Fig. 6a). As shown in Fig. 6b, immunization of VP1/CRA resulted in regression of CMS5-VP1 tumors but incapable to retard CMS5 nor 4T1 tumor growth. Thus, antitumor response induced by VP1/CRA was VP1-specific, neither mediated bystander’s effect nor adjuvant effect. These results provided a piece of convincing evidence that activation of tumor antigen-specific T cells is vital for antitumor activity. Thus, the vaccine formulated by VP1 and CRA might be a potential approach to eradicate MCC.

## Discussion

MCC is rare but being taken notice of owing to increased incidence and high risk of recurrence^22^. Rejuvenation of CD8^+ ^T cells has played an essential role in MCC survival since immunotherapies with anti-PD-L1 or anti-PD-1 have brought better outcomes and prognosis in patients with MCC. No MCV antigen-specific immunotherapeutic approach has been developed, although oncoprotein target vaccines have been shown to induce neoepitope-specific T-cell response in immunogenic tumor^23^. One of the obstacles was that no suitable animal model available to evaluate such vaccine efficacy since MCC tumor cells neither grow in vitro nor MCV infectivity in cultured cells. Adopting the strategy of TA-expressing tumor-bearing mice generated with ST- or LT-expressing B16 melanomas cells to evaluate DNA vaccines targeting T antigen^12,13^, we established a murine CMS5 tumor cell line expressing MCV capsid protein VP1. MCV therapeutic vaccine candidates targeted MCV capsid protein VP1 was formulated with various adjuvant compositions, including GIA, or CA, or RA, or MA, or CRA, respectively. Although all these candidates showed some degrees of antitumor effects, combining VP1 and CRA had achieved a complete antitumor efficacy. Such combination induced significantly antitumor effects and lead to durable tumor regression. Triple treatment of such variety could evoke marked long-lasting antitumor effects to inhibit the rechallenged CMS5-VP1 tumor cells. This study demonstrated that therapeutic vaccine target MCV capsid protein VP1 might potentially cure the MCC.

The VP1 is essential for serological diagnosis, and epidemiologic studies showed 79–96% seroprevalence among adulthood^7^. Furthermore, a higher titer of anti-VP1 antibodies resulted in increased overall survival and a lower probability of recurrence in patients with MCC^14^. Although the VP1 of MCV as a target for MCC, several important studies have demonstrated that VP1 seems not to be expressed in the MCC tumors^24,25^. In fact, about 23% of MCC patients were MCV-negative but with seropositive antibodies against MCV capsid protein VP1^26^. It may be due to the expression of the VP1 gene along with other oncogenic genes, including large T within the MCV virus, to trigger infected cells to become MCC through lengthy carcinogenesis. Those viral genes or gene products are no longer necessary beyond that point once cancer started to grow. Several similar cases of viral-driven tumors lost expressions of their capsule antigen, including HPV-related cervical cancers, HBV-related hepatitis carcinomas, and Kaposi’s sarcoma induced by human herpesvirus 8, summarized in a review^27^. An example from the HBV and HCC, a chronic HBV infection, defined by HBsAg positivity for more than six months, can significantly increase the risk of developing liver disease, including chronic hepatitis, cirrhosis, and HCC. It has been reported that the level of HBsAg expressions in HCC patients was reduced in most HCC tissues, only 10.7% of HCC tissues were detectable of HBsAg^28^. This notion is further supported by recent observations in the association of MCV and MCC. For instance, the MCV particles were detectable in primary MCC tumor cell cytoplasm and nuclei, and the VP1 gene was detectable in primary MCCs, but lost in metastatic tumors^29,30^. Phylogenetic VP1 study revealed that mutations in VP1 might impact viral DNA integration and tumorigenesis through protein folding, membrane binding, or antibody escape^31–33^. Most importantly, VP1-derived epitopes elicit CD8^+ ^T-cell responses have been reported^15,34^, and VP1-specific CD4^+ ^T-helper (Th) cell responses were found both in seropositive and seronegative healthy individuals but lacking in patients with MCC. Therefore, VP1 as an immunotherapeutic target may play a major role in specific cell-mediated immunity in surveillance of MCV infection and tumorigenesis in patients with MCC^34,35^.

TA is also a target for such an approach. However, TA-targeted DNA vaccines in the preclinical study have been reported to activate antigen-specific T-cell response, which is vital for tumor clearance^12,13^. Administration of TA in humans might raise safety concerns due to its potential activity of tumorigenesis.

Both CD4^+ ^and CD8^+ ^T cells played a functional role herein since the antitumor response was entirely lost upon depletion of CD4^+ ^and CD8^+ ^T cells. This result demonstrated that both CD4^+ ^and CD8^+ ^T cells responses elicited by VP1/CRA are crucial for tumor regression. Interestingly, adjuvant of RA or CRA without antigen has shown little antitumor efficacy, as showed in Figs. 1 and 3, indicating that the antitumor effects of VP1/CRA were antigen-specific. The antigen-specific antitumor activity was further confirmed in Fig. 6, in which no antitumor results occurred in CMS5 or 4T1 challenge tumor model without VP1 expression after the vaccinations of VP1/CRA.

The immunotherapy effect might be abated by the immunosuppressive environment created by many tumors. Virtually, most MCC patients are in older adults, and immunocompromised, highly antigenic MCV-related MCC tumors could escape from host immune clearance by inducing tolerogenic microenvironment, including a high level of Tregs and upregulation of PD-L1 expression^7^. Adjuvants derived from Toll-like receptor (TLR) agonists can break the tolerogenic responses. Single TLR agonist seems not to be effective, but two of them adopted as an adjuvant system had an immune synergy to enhance antigen-specific T-cell responses and achieved the most effective tumor regression observed in this study. R848 (Imiquimod) has been approved to treat basal cell carcinoma and precancerous lesions such as actinic keratosis and induce cytokine secretion, macrophage activation, and enhancement of cellular immunity^20,36,37^. CpG as the TLR9 agonist has been reported to activate tumor-specific CD8^+ ^T cells or modulate the tumor microenvironment by down-regulating Treg or MDSC levels^19,38,39^. FDA had approved a hepatitis B vaccine adjuvanted with CpG in 2017^40^. T cells from tumor-bearing mice immunized with VP1/CA or VP1/RA shown increased secretion of IL-2, IFN-*γ*, and TNF-*α* than T cells from untreated tumor-bearing mice in this study, which were correlated with in vivo antitumor effects induced by VP1/CA or VP1/RA. Remarkably, the VP1 in a combination of both adjuvants caused more cytokine secretions and ultimately tumor regression, which was associated with reduced frequency of TGF-β-expressing CD4^+ ^Treg cells. Although more studies were required to elucidate the capacity of downregulation of immunosuppressive factors and correlation with the regression, vaccination with VP1/CRA greatly impacted antitumor cellular immune responses and presented a feasible approach to treat patients with MCC.

In summary, the vaccine of VP1/CRA developed in this study was the first therapeutic vaccine to target the capsid protein. More importantly, triple therapy with the vaccine could induce more robust antitumor efficacy in the murine CMS5-VP1 model. Using this model, we demonstrated that treatment with VP1/CRA could elicit a potent immune response and long-lasting effect by inducing a VP1-specific cellular immune response. Although we developed a VP1-targeting therapeutic vaccine in the current study, more studies are needed in future clinical investigations, including patients with MCC for VP1 positivity screenings before using such treatments.

## Materials and Methods

### Expression and purification of the recombinant MCV VP1 protein

The DNA encoding full-length of MCV major capsid protein VP1 was codon-optimized and synthesized by GenScript (Nanjing, Jiangsu, China) and cloned into a pET28a plasmid (Invitrogen, Carlsbad, CA, USA). The plasmid pET28a-VP1 was transformed into *E.Coli* strain Rosseta (TIANGEN BIOTECH, Beijing, China). The positive colonies of transformed *E.Coli* were selected and cultivated in 10 ml LB medium (yeast extract 5 g/l, tryptone 10 g/l, NaCl 5 g/l) supplemented with 50 µg/ml Kanamycin at 37 ˚C and at 250 rpm/min in a shaker. When the OD_600_ value reached 1–2, the culture was transferred into 1 l flasks containing 500 ml of fermentation medium (yeast extract 24 g/l, tryptone 12 g/l, K_3_PO_4_ 13.79 g/l, MgSO_4_ 0.12 g/l, 50 µg/ml Kanamycin) and grown at 37 ˚C and shaking at 250 rpm/min for 3 h before this culture was inoculated a 10 l fermenter with 5 l fermentation medium (tryptone 10 g/l, yeast extract 6.4 g/l, (NH_4_)_2_SO_4_ 0.88 g/l, sodium citrate 0.98 g/l, ammonium ferric citrate 0.083 g/l, glucose 10 g/l, KH_2_PO_4_ 6.25 g/l, K_2_HPO_4 _∙ 3H_2_O 12.5 g/l, CaCl_2 _∙ 2H_2_O 16.6 mg/ml, ZnSO_4 _∙ 7H_2_O 18.26 mg/l, MnSO_4 _∙ H_2_O 4.15 mg/l, CuSO4 ∙ 5H_2_O 11.62 mg/l, ammonium molybdate tetrahydrate 0.83 mg/l, sodium tetraborate decahydrate 16.6 µg/l). Fermentation was carried out in pH-Stat fed-batch mode, maintaining the pH at 7.0. After three h of cultivation, as the OD_600_ reached 30, IPTG was added with a final concentration of 0.5 mM to induce the expression of recombinant VP1 protein at 30 ˚C for 4 h. The broth was harvested and centrifuged at 5000 x g for 30 min at 4 ˚C to collect the cell pellet. The cell pellet was resuspended with washing buffer (0.1 M NaH_2_PO_4_, 20 mM Tris, 0.3 M NaCl, pH 7.0) and homogenized by a high-pressure homogenizer (ATS, Jiangsu, China) followed by centrifuging at 8000 x *g* for 30 min at 4 ˚C to collect the inclusion bodies. The inclusion bodies were dissolved in denatured buffer (0.1 M NaH_2_PO4, 20 mM Tris, 0.3 M NaCl, 8 M Urea, 0.1% Trixon-100) and agitated 4 ˚C overnight and harvested by centrifugation at 8000 x *g* for 15 min at 4 ˚C. The denatured inclusion bodies were passed through a 0.45 µm filter membrane (Millipore, Billerica, USA) and loaded onto a 20 ml HisTrap FF column (Ni Chelating Sepharose Fast flow, GE Healthcare, Fairfield, USA) that equilibrated with Buffer I (0.1 M NaH_2_PO_4_, 20 mM Tris, 0.3 M NaCl, 8 M Urea, pH 7.0). The unbound proteins were washed with Buffer II (0.1 M NaH_2_PO_4_, 20 mM Tris, 0.3 M NaCl, 8 M Urea, 20 mM Imidazole, pH 7.0). The bound proteins were eluted with Buffer III (0.1 M NaH_2_PO_4_, 20 mM Tris, 0.3 M NaCl, 8 M Urea, 500 mM Imidazole, pH 7.0). The eluted recombinant protein under denaturing conditions was subjected to an SDS-PAGE and western blot analysis with mouse anti-His polyclonal antibody (Invitrogen, USA) as the 1st antibody at 1:2500 dilution and HRP-conjugated Goat anti-mouse IgG (Bio-Rad, CA, USA) as the 2nd antibody at 1:5000 dilution. The denatured protein was stepwise dialyzed at 4 ˚C to remove imidazole and urea and disassembled by adding 10 mM DTT and 10 mM EGTA at 20 ˚C for 2 h, followed by dialysis in Buffer VIII (50 mM Tris, 0.8 M (NH_4_)_2_SO_4_, 0.2 M NaCl, 0.5 mM GSH, 4.5 mM GSSG, 2 mM CaCl_2_, 5%(v/v) glycerol, pH 6.4) to allow VP1 to reassemble into VLP. After 48 h, the buffer was changed to Buffer IX (50 mM Tris, 0.2 M NaCl, 5%(v/v) glycerol, pH 7.0). The reassembled protein was passed through a 0.22 µm filter membrane (Millipore, Billerica, USA), and the concentration was determined by BCA protein assay kit (Thermo Fisher, USA). The reassembled protein was stored at −80 ˚C until use.

### Cells

CMS5 (murine sarcoma cells) were kindly provided from Dr. You-yong Lu, Beijing Cancer Hospital, Cancer Research Institute, China) and 4T1 (murine mammary tumor cells) were purchased from ATCC. CMS5 and 4T1 were cultured in RPMI 1640 medium (Gibco, NY, USA) supplemented with 10% FBS (Gibco, NY, USA) and 1% antibiotics (Gibco, NY, USA). To generate a VP1-expressing tumor cell line, the lentivirus encoding VP1 was codon-optimized and synthesized by GenScript (Nanjing, Jiangsu, China), then cloned into the vector pcDH-GFP-Puro (gifted by Shibo Jiang laboratory, Fudan University). The plasmid pcDH-VP1 was transfected into CMS5 using lipofectamine (Invitrogen, Carlsbad, USA) and cultivated in RPMI 1640 medium supplemented with 10% FBS in CO_2_ incubator at 37^o^C for 48 h. Puromycin (YEASEN, Shanghai, China) was added into the culture with a final concentration of 10 µg/ml. Cytotoxicity of puromycin resulted in the complete death of CMS5 cells and survival of CMS5-VP1 cells. A single clone of CMS5-VP1 cells was selected and propagated in RPMI 1640 medium supplemented with 10% FBS and 10 µg/ml Puromycin. Flow cytometry and Western Blot analysis were performed to determine VP1 expressed in CMS5-VP1 cells.

### Tumor model and VP1 analysis

To generate the VP1-expressing tumorigenic model, various VP1-expressing tumor cells CMS5-VP1 were subcutaneously inoculated into 6–8 weeks BALB/c mice from 0.5–3 × 10^6^, and tumor growth was monitored. As a result, the 1 × 10^6^ of CMS5-VP1 inoculation was selected. For detection of VP1-expressing in a CMS5-VP1 tumor model, both CMS5-VP1 and CMS5 tumors were removed and cut into pieces, followed by incubating with trypsin at 37 ˚C with 5% CO_2_ for 30 min, the cells suspension were separated, and cell lysis was loaded and separated by 4–12% SDS-PAGE (SANGON, Shanghai, China). The protein was transferred onto the polyvinylidene fluoride (PVDF) membrane (Bio-Rad, CA, USA). The PVDF membrane was blocked with phosphate-buffered saline-Tween20 (PBST) containing 5% skim milk at room temperature and probed with rabbit anti-VP1 polyclonal antibody at 1:5000 dilution in PBST containing 2% skim milk for 1 h at room temperature, followed by incubation with HRP-conjugated Goat anti-Rabbit IgG (Bio-Rad, CA, USA) at 1:10,000 dilution in PBST containing 2% skim milk after washed three times with PBST. The signal was developed using a High-signal ECL Western Blotting Substrate (Tanon, Shanghai, China) after washed five times with PBST.

### Mice

Female BALB/c mice (6–8 weeks) were purchased from SINO-BRITISH SIPPR/BK LAB ANIMAL Ltd. (Shanghai, China) and maintained under specific pathogen-free conditions. All animal experiments were approved by the Committee of Experimental Animals of SHMC with the following reference number: 20160225–115.

### Formulation of MCV therapeutic vaccine candidates

The purified recombinant VP1 protein was adsorbed with Al(OH)_3_ (10 mg/ml, Brenntag Biosector, Denmark) at the volume of 1:1, and further formulated with several adjuvants in this study. Adjuvant GIA is composed of GM-CSF (North China Pharmaceutical, China), IFN-α (SinoBiological, China), and Al(OH)_3_. CA was composed with CpG1826 (Generay, China) and Al(OH)_3_, RA with R848 (Invivogen, USA) and Al(OH)_3_, CRA with CpG1826, R848, and Al(OH)_3_, MA with MPL (Institute of Medical Engineering, China) and Al(OH)_3_, A with Al(OH)_3_. Each vaccination contains 10 µg VP1, 500 µg Al(OH)_3_ with either of 10 µg GM-CSF and 1 µg IFN-α, or 10 µg CpG1826, or 10 µg R848 (Invivogen, USA), or 50 µg MPL or a combination of 10 µg CpG1826 and 10 µg R848.

### In vivo antitumor effects

CMS5-VP1 tumor cells were subcutaneously inoculated into the left flank back of BALB/c mice at day 0. Tumor-bearing mice were grouped randomly on day 5 when the CMS5-VP1 tumors were palpable. For vaccination studies, animals were vaccinated intramuscularly at one-week intervals. For rechallenge studies, mice with tumor eradicated for 30 days were rechallenged with 1 × 10^6^ of CMS5-VP1 tumor cells in the right flank back. For the antigen-specificity study, BALB/c mice were subcutaneously inoculated with a 1 × 10^6^ of CMS5-VP1 tumor cells, wild-type CMS5 tumor cells, and 4T1 tumor cells at day 0, and vaccinated at day 5 with one-week intervals. To evaluate T cells’ role in antitumor effects generated by the therapeutic vaccine, anti-CD3, anti-CD4 or anti-CD8 monoclonal antibodies (Bioxcell, NH, USA) were injected into CMS5-VP1 tumor-bearing mice to deplete T cells followed by vaccination 24 h later. Tumor growth was measured every 2 or 3 days after tumor rechallenge.

### Tumor size measurement

Tumor growth was monitored by visual inspection and palpation. In addition, tumor size was measured by digital caliper every 2 or 3 days. The tumor volume was calculated using the formula “length × width^2^ × 0.5”.

### Flow cytometry

CMS5-VP1 or CMS5 cells (1.5 × 10^6^) were washed twice with PBS, then incubated with eFluor780 conjugated FVD (eBioscience, OR, USA) in the dark at room temperature for 15 min to distinguish live and dead cells. After washing and permeabilized by Fixation/Permeabilization buffer (BD), cells were washed and incubated with 500-folds diluted mouse anti-VP1 sera at room temperature for 1 h. Then, cells were washed and stained with FITC-conjugated Rat anti-mouse IgG (Biolegend, CA, USA) at room temperature for 1 h. Finally, stained CMS5 or CMS5-VP1 cells were washed twice and resuspended in 200 µl PBS. Both stained CMS5 or CMS5-VP1 cells were analyzed for VP1 expressing by a Canto II Flow Cytometer (BD, CA, USA) and data analyzed by FlowJo software (BD, CA, USA).

Flow cytometry and intracellular cytokine staining to determine the antigen-specific T-cell responses induced by vaccines was performed. Briefly, splenocytes isolated from vaccine immunized CMS5-VP1 tumor-bearing mice were seeded into each well with 1.5 × 10^6^/well and stimulated with 2 µg of VP1, PMA (50 ng/ml) /Ionomycin (1 µg/ml) (BD, CA, USA) as the positive control. Cells were stimulated at 37 ˚C with a 5% CO_2_ incubator for 16 h, followed by blocking with 1 µg/ml of Brefedlin A (BD, CA, USA) for another 4 h. Stimulated cells were surfaced stained with FVD-eFluor780 (eBioscience, OR, USA), anti-CD3-eFluor506 (17A2, eBioscience, OR, USA), anti-CD4-FITC (GK1.5, BD, CA, USA), anti-CD8-PE (53–6.7, BD, CA, USA) for 15 min in dark at room temperature, then washed and permeabilized with Fixation/Permeabilization buffer (BD, CA, USA). After surface staining, the cells were intracellular stained with anti-IL-2-Percp/Cy5.5 (JES6-5H4, BD, CA, USA), anti-TNF-*α*-APC (MP6-XT22, Biolegend, CA, USA), anti-IFN-*γ*-BV421 (XMG1.2, Biolegend, CA, USA) for 30 min at 4 ˚C. Cells were washed twice and then resuspended with 200 µl FBS.

For Treg staining, lymphocytes from lymph node were surface stained with eFluor780 conjugated FVD (eBioscience, OR, USA), anti-CD3-eFluor506 (17A2, eBioscience, OR, USA), anti-CD4-FITC(GK1.5, BD, CA, USA), and anti-CD8-PE (53-6.7, Biolegend, CA, USA) for 15 min at room temperature, following fixation and permeabilization with Transcription Factor Buffer Set (Biolegend, CA, USA), the cells were stained with anti-FOXP3-BV421(MF-14, Biolegend, CA, USA) and anti-LAP-PE/Cy7 (TW7-16B4, eBioscience, OR, USA) for 30 min at room temperature. Cells were washed twice with Perm buffer and then resuspended with 200 µl PBS. The stained cells were analyzed by a Canto II flow cytometer (BD, CA, USA) and FlowJo software.

### Enzyme-linked immunosorbent assay (ELISA)

Veinous blood was collected from mice sacrificed and used to detect anti-VP1 antibody titers by ELISA. ELISA was performed using 96-well assay plates (Costar, NA, USA) coated with VP1 protein (100 ng/well) and incubated at 37 ˚C for 1 h. After the antigen solution was removed, each well of the plates was blocked for 1 h at 37 ˚C with 300 µl PBST containing 5% skim milk. Serums were diluted in PBST containing 2% skim milk. Serum samples were subjected to a series of ten twofold dilutions (range 1:400 to 1:2.0 × 10^6^). The blocking solution was removed, followed by added diluted serum samples to the antigen-coated plates. The plates were incubated at 37 ˚C for 1 h followed by washing four times with PBST. HRP-conjugated goat anti-mouse IgG (BioRad, CA, USA) was diluted a 1:5000 in PBST containing 2% skim milk, and 100 µl of the dilution was added to each well. The plates were incubated at 37 ˚C for 1 h and then washed as described above. Freshly prepared TMB hydrogen peroxide solution was added at 100 µl per well. The enzyme reaction was incubated at 37 ˚C in the dark for 5 min and stopped by the addition of 50 µl of 2 M H_2_SO_4_ solution. The plates were read at 450 nm in an automated microtiter plate reader(Molecular Devices, CA, USA) with a reference wavelength of 620 nm. The mean OD (optical density) of serums from mice vaccinated PBS multiple 2.1 was defined as the cutoff point for seropositivity. By using the cutoff point, serology results were defined as antibody positive and negative.

### Statistical analysis

Data were presented as mean ± standard error of the mean (SEM). Differences among and between groups were determined by ordinary one-way ANOVA and Mann–Whitney test, respectively. A *p* < 0.05 value was considered significantly different statistically.

### Reporting summary

Further information on research design is available in the Nature Research Reporting Summary linked to this article.

## Supplementary information

Supplementary Information

Reporting Summary

## Data Availability

The data that support the findings of this study are available from the corresponding author upon reasonable request.

## Code availability

This study did not generate any unique code.
