# Supplementary material for: Development of a therapeutic vaccine targeting Merkel cell polyomavirus capsid protein VP1 against Merkel cell carcinoma
Source: NPJ Vaccines. 2021 Oct 5;6:119. doi: 10.1038/s41541-021-00382-9 (PMC8492671; doi:10.1038/s41541-021-00382-9)
Supplement: Supplementary file 1 — Supplementary Information [file 41541_2021_382_MOESM1_ESM.pdf]

Supplementary Data

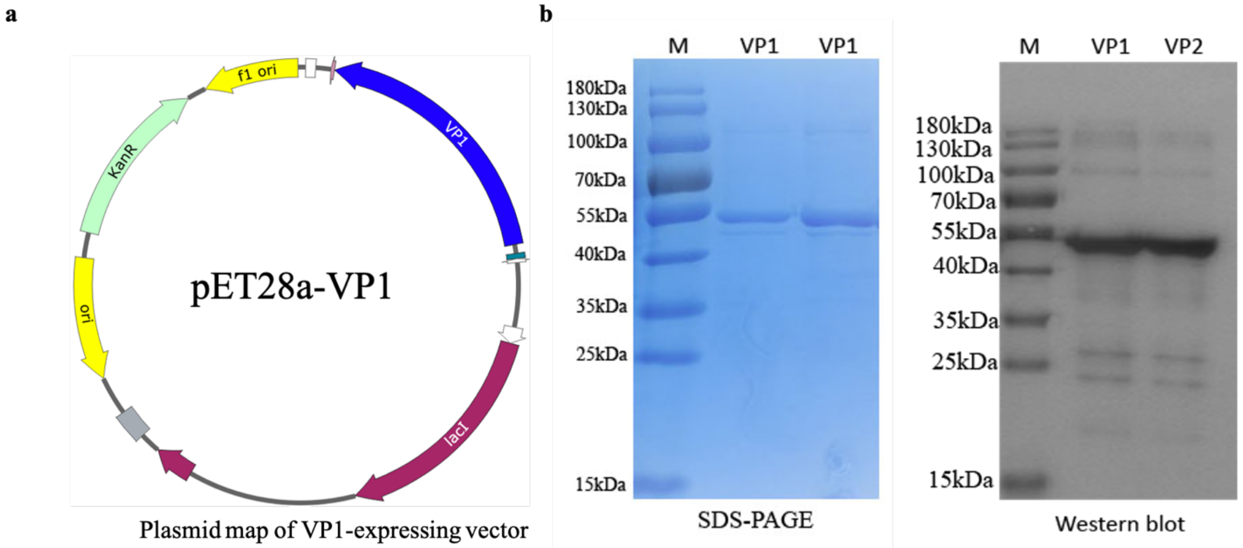

Supplementary Figure 1. Expression vector and production of VP1.

(a) Map of VP1 expressing vector pET28a-VP1. Gene encoding VP1 was synthesized and cloned into pET28a plasmid and then expressed in an E.*coli* protein expression system as described in materials and methods. **(b)** SDS-PAGE and Western Blot of purified VP1. In brief, the expressed VP1 protein was purified by Ni Chelating Sepharose and then analyzed by SDS-PAGE (left panel) and Western Blot (right panel). The VP1 protein was separated by SDS-PAGE on a 12% gel followed by staining with Coomassie Blue. An equal amount of protein was analyzed by Western Blot using rabbit anti-VP1 polyclonal antibody as 1st antibody, and HRP conjugated Goal anti-Rabbit IgG as 2nd antibody to identify the target protein. Samples were derived from the same experiment, SDS-PAGE and Western blot were processed in parallel.

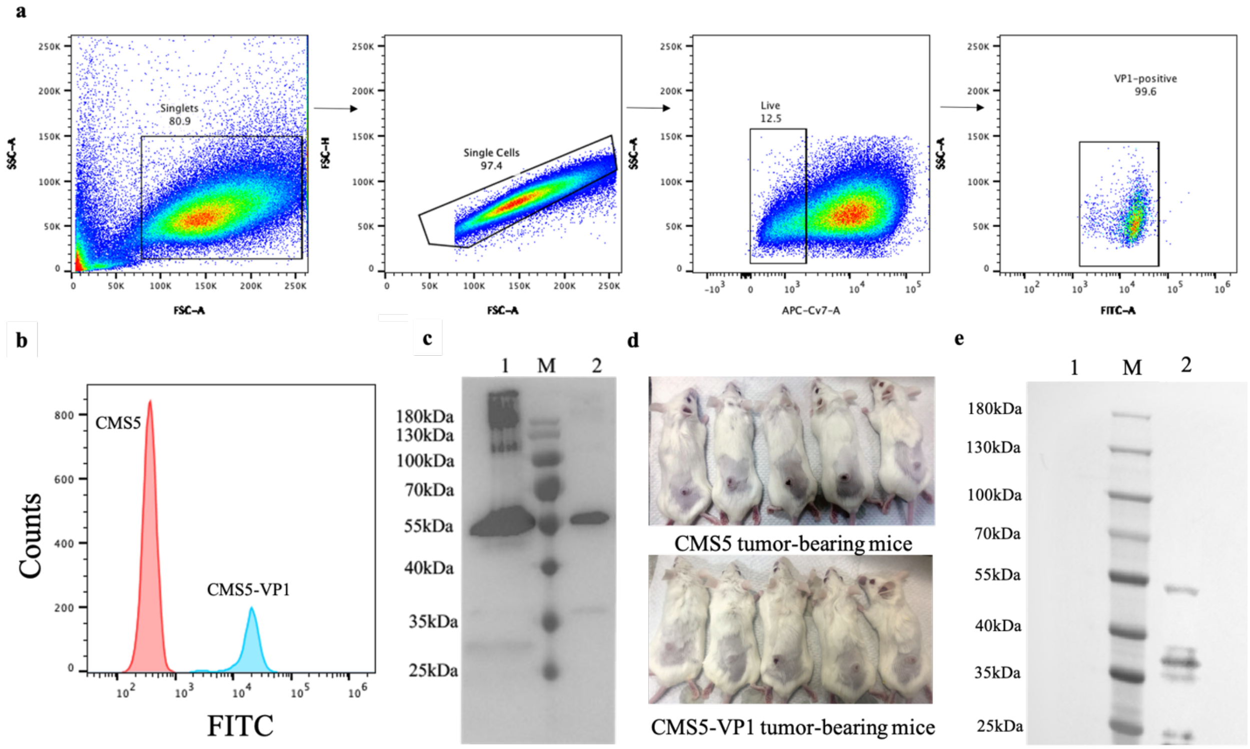

Supplementary Figure 2. Generation and characterization of VP1-expressing CMS5-VP1 tumor cell line.

CMS5 cells were transduced with a plasmid pcDH-VP1 containing a codon-optimized gene encoding the capsid protein VP1 of Merkel cell polyomavirus under control of a CMV promoter to generate a tumorigenic VP1-expressing cell line, CMS5-VP1. Expression of VP1 by CMS5-VP1 cells were detected using FACS and Western Blot. Samples were derived from the same experiment, and Western blot were processed in parallel.

(a) Gating strategy for FACS of VP1-expressing CMS5-VP1 cells. As singlets were gated, lived cells were gated for detection of VP1-expression with mouse anti-VP1 sera as 1st and FITC conjugated anti-mouse IgG as 2nd antibody.

(b) Characterization of transduction of CMS5-VP1 cells. CMS5-VP1 cells (Blue line, filled blue histogram) or control CMS5 cells (red line, filled red histogram) were characterized for VP1

expression by flow cytometry analysis using the gating strategy above. In brief, CMS5 or CMS5-VP1 cells (1.5×10^6) were stained with eFluor780 conjugated FVD for live and dead cells, followed by inoculating with mouse anti-VP1 sera, then washed with PBS and stained with FITC conjugated Rat anti-Mouse IgG. Finally, stained CMS5 or CMS5-VP1 cells were analyzed by flow cytometer.

(c) Characterization of VP1 expression by Western Blot. Briefly, CMS5-VP1 cells (1×10^6) were treated with cell lysis buffer and detected for VP1 expressing by Western blot with purified VP1 as a positive control. The membrane was probed with Rabbit anti-VP1 polyclonal antibody followed by HRP conjugated Goat anti-Rabbit IgG. *Lane 1, purified VP1. Lane 2, CMS5-VP1 cells. Lane M, pre-stained protein marker.*

(d) Tumorigenicity of CMS5-VP1. Briefly, naïve BALB/c mice (5 per group) were inoculated with CMS5-VP1 or CMS5 tumor cells (1×10^6 per mouse) subcutaneously to generate the CMS5-VP1 or CMS5 tumor-bearing mice to observe tumor growth until day 20 post tumor inoculation.

(e) Characterization of VP1 expression in CMS5-VP1 tumors from above (C). Briefly, both CMS5-VP1 and CMS5 tumors were excised and treated with trypsin to separate tumor cells. Equal cells were treated with cell lysis buffer. Finally, a Western blot was performed to detect VP1 expressed in CMS5 or CMS5-VP1 tumors. The membrane was probed with Rabbit anti-VP1 polyclonal antibody followed by HRP conjugated Goat anti-Rabbit IgG. *Lane 1, CMS5 tumor. Lane 2, CMS5-VP1 tumor. Lane M, pre-stained protein marker.*

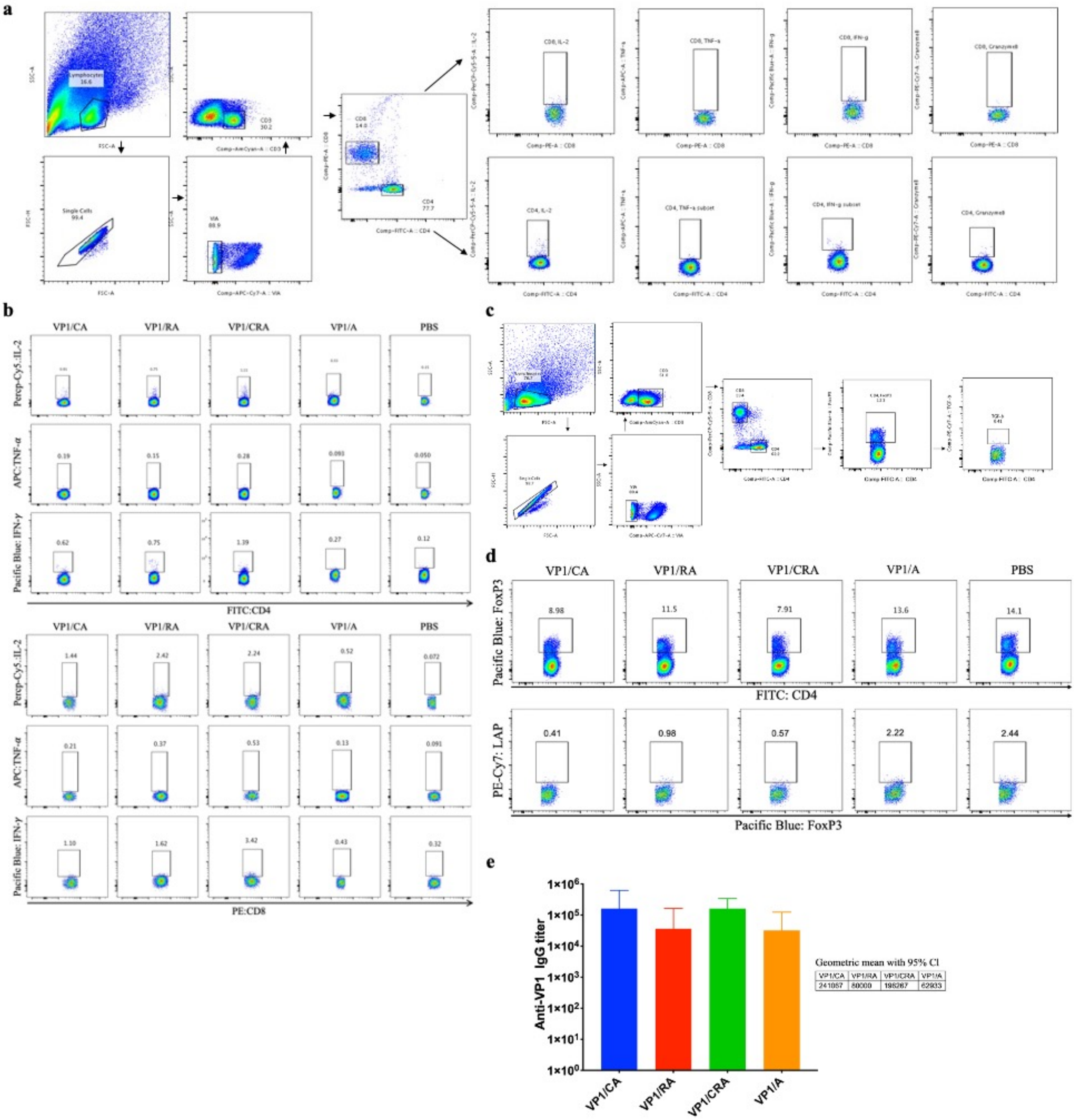

Supplementary Figure 3. Responses of multi-functional T cells and antibodies induced by Triple-treatment of VP1/CRA

(a) Gating strategy for FACS of effective CD4^{+} and CD8^{+} T cells from tumor-bearing mice treated with VP1/CRA, VP1/CA, VP1/RA, VP1/A, or PBS. Briefly, gated lymphocytes were gated for single, lived cells with FVD-eFluor780, followed by gating CD3^{+} T cells with the anti-CD3-

eFluor506. Then $CD3^+CD4^+$ and $CD3^+CD8^+$ T cells were gated with anti-CD4-FITC or anti-CD8-PE for detection of expression of IL-2, IFN-γ or TNF-α.

(b) Flow cytometry for intracellular markers of effective $CD4^+$ and $CD8^+$ T cells from tumor-bearing mice treated with VP1/CRA, VP1/CA, VP1/RA, VP1/A, or PBS as gating strategy shown in **(a)**. Briefly, spleens were removed from vaccinated mice for the separation of splenocytes. Splenocytes were stimulated with 2μg purified VP1 for 20h, with ION and PMA as positive stimuli. Live, $CD3^+CD4^+$ or $CD8^+$ T cells were gated and graphed for IL-2, TNF-α, and IFN-γ expression (representative data shown).

(c) Gating strategy for FACS of FOXP3-expressing $CD4^+$ Tregs in inguinal lymph nodes of tumor-bearing mice vaccinated VP1/CRA, VP1/CA, VP1/RA, VP1/A, or PBS. Briefly, gated lymphocytes were gated for single, lived cells with FVD-eFluor780, followed by gating $CD3^+$ T cells with the anti-CD3-eFluor506. Then $CD3^+CD4^+$ were gated with anti-CD4-FITC and anti-CD8-PE. Subsequently, $CD3^+CD4^+$ Tregs with FOXP3-expressing cells were gated with anti-FOXP3-BV421, and expression of TGF-β by $CD3^+CD4^+$ Tregs were gated by anti-LAP-PE/Cy7.

(d) Flow cytometry analyzed FoxP3-expressing $CD4^+$ Tregs in inguinal lymph nodes of tumor-bearing mice vaccinated VP1/CRA, VP1/CA, VP1/RA, VP1/A, or PBS as gating strategy shown in **(c)**. Briefly, lymphocytes were separated from inguinal lymph nodes near left hind limps, then live $CD3^+CD4^+$ $FoxP3^+$ T cells were gated and graphed for TGF-β expression with the marker LAP (surface latency-associated peptide of TGF-β) (representative data shown).

(e) ELISA analyzed serum samples from tumor-bearing mice treated with VP1/CRA, VP1/CA, VP1/RA, VP1/A, A, or PBS as mentioned in Materials and Methods. Series diluted sera were incubated in VP1-coated plates with 2 repetitions at 37˚C for 1h, followed by incubating with diluted HRP conjugated goat anti-mouse IgG at 37˚C for 1h. Then enzyme reactions were

performed and stopped by 2M H_2SO_4 solution. The optical density (OD) was read, and the antibody titers were defined with 2.1 multiple cutoff points.
