# Supplementary material for: Development of a therapeutic vaccine targeting Merkel cell polyomavirus capsid protein VP1 against Merkel cell carcinoma
Source: NPJ Vaccines. 2021 Oct 5;6:119. doi: 10.1038/s41541-021-00382-9 (PMC8492671; doi:10.1038/s41541-021-00382-9)
Supplement: Supplementary file 2 — Reporting Summary [file 41541_2021_382_MOESM2_ESM.pdf]

nature portfolio

Corresponding author(s): Bin Wang
NPJVACCINES-01251R

Last updated by author(s): Jun 29, 2021

Reporting Summary

Nature Portfolio wishes to improve the reproducibility of the work that we publish. This form provides structure for consistency and transparency in reporting. For further information on Nature Portfolio policies, see our Editorial Policies and the Editorial Policy Checklist.

Statistics

For all statistical analyses, confirm that the following items are present in the figure legend, table legend, main text, or Methods section.

n/a	Confirmed	
☐	☒	The exact sample size (*n*) for each experimental group/condition, given as a discrete number and unit of measurement
☐	☒	A statement on whether measurements were taken from distinct samples or whether the same sample was measured repeatedly
☐	☒	The statistical test(s) used AND whether they are one- or two-sided<br>*Only common tests should be described solely by name; describe more complex techniques in the Methods section.*
☐	☒	A description of all covariates tested
☐	☒	A description of any assumptions or corrections, such as tests of normality and adjustment for multiple comparisons
☐	☒	A full description of the statistical parameters including central tendency (e.g. means) or other basic estimates (e.g. regression coefficient) AND variation (e.g. standard deviation) or associated estimates of uncertainty (e.g. confidence intervals)
☐	☒	For null hypothesis testing, the test statistic (e.g. *F*, *t*, *r*) with confidence intervals, effect sizes, degrees of freedom and *P* value noted<br>*Give P values as exact values whenever suitable.*
☒	☐	For Bayesian analysis, information on the choice of priors and Markov chain Monte Carlo settings
☒	☐	For hierarchical and complex designs, identification of the appropriate level for tests and full reporting of outcomes
☐	☒	Estimates of effect sizes (e.g. Cohen's *d*, Pearson's *r*), indicating how they were calculated

Our web collection on statistics for biologists contains articles on many of the points above.

Software and code

Policy information about availability of computer code

Data collection	Microsoft Excel, FlowJo
Data analysis	GraphPad Prism 8

For manuscripts utilizing custom algorithms or software that are central to the research but not yet described in published literature, software must be made available to editors and reviewers. We strongly encourage code deposition in a community repository (e.g. GitHub). See the Nature Portfolio guidelines for submitting code & software for further information.

Data

Policy information about availability of data

All manuscripts must include a data availability statement. This statement should provide the following information, where applicable:
- Accession codes, unique identifiers, or web links for publicly available datasets
- A description of any restrictions on data availability
- For clinical datasets or third party data, please ensure that the statement adheres to our policy

The data that support the findings of this study are available from the corresponding author upon reasonable. This study did not generate any unique code.

Field-specific reporting

Please select the one below that is the best fit for your research. If you are not sure, read the appropriate sections before making your selection.

☒ Life sciences ☐ Behavioural & social sciences ☐ Ecological, evolutionary & environmental sciences

For a reference copy of the document with all sections, see nature.com/documents/nr-reporting-summary-flat.pdf

Life sciences study design

All studies must disclose on these points even when the disclosure is negative.

Sample size	All animal experiments were performed with 5-6 mice/group, all data were analyzed using One-way ANOVA or Two-way ANOVA
Data exclusions	No data was excluded in any experiments.
Replication	All experiments were repeated twice or thrice, all data could be reproduced.
Randomization	All experiment animals including tumor-bearing mice were divided into groups randomly.
Blinding	This study is a preclinical study to investigate a therapeutic vaccine, blinding is not necessary.

Reporting for specific materials, systems and methods

We require information from authors about some types of materials, experimental systems and methods used in many studies. Here, indicate whether each material, system or method listed is relevant to your study. If you are not sure if a list item applies to your research, read the appropriate section before selecting a response.

Materials & experimental systems

n/a	Involved in the study	
☐	☒	Antibodies
☐	☒	Eukaryotic cell lines
☒	☐	Palaeontology and archaeology
☐	☒	Animals and other organisms
☒	☐	Human research participants
☒	☐	Clinical data
☒	☐	Dual use research of concern

Methods

n/a	Involved in the study	
☒	☐	ChIP-seq
☐	☒	Flow cytometry
☒	☐	MRI-based neuroimaging

Antibodies

Antibodies used	eFlour780 FVD(eBioscience, #65-0865-14), anti-CD3-eFluor506 (eBioscience, #69-0032-82, Clone: 17A2), anti-CD4-FITC(BD, # 553047, Clone: GK1.5), and anti-CD8-PE (BD, #553033, Clone: 53-6.7), anti-IL-2-Percp/Cy5.5 (BD, #560544, Clone: JES6-5H4), anti-TNF-α-APC (Biolegend, #506308, Clone: MP6-XT22), anti-IFN-γ-BV421 (Biolegend, #505803, Clone: XMG1.2), anti-FOXP3-BV421(Biolegend, #126419, Clone: MF-14) and anti-LAP-PE/Cy7 (eBioscience, #25-9821-80, Clone: TW7-16B4), FITC-conjugated Rat anti-mouse IgG (Biolegend, #406001, Clone: B249772), InVivoMab anti-mouse CD3e(BioXcell, #BE0001-1, Clone: 145-2C11), InVivoMab anti-mouse CD4(BioXcell, #BE0119, Clone: YTS 191), InVivoMab anti-mouse CD8(BioXcell, #BE0061, Clone: 2.43), Goat Anti-Mouse IgG(H+L)-HRP conjugate(BioRad, #1706516), Goat Anti-Rabbit IgG(H+L)-HRP conjugate(BioRad, #1706515)
Validation	Several repeated tests for antibodies were performed with positive and negative controls for insure their qualities.

Eukaryotic cell lines

Policy information about cell lines

Cell line source(s)	4T1 mammary tumor cells(CRL-2539™) were puchased from ATCC. CMS5 murine sarcoma cells were kindly provided from Dr You-yong Lu, Beijing Cancer Hospital, Cancer Research Institute, China
Authentication	N/A
Mycoplasma contamination	N/A
Commonly misidentified lines (See ICLAC register)	N/A

Animals and other organisms

Policy information about studies involving animals; ARRIVE guidelines recommended for reporting animal research

Laboratory animals	BALB/c female mice aged 6-8weeks were used.
Wild animals	N/A
Field-collected samples	All BALB/C mice involved in the study were maintained under specific pathogen-free conditions.
Ethics oversight	All animal experiments were approved by the Committee of Experimental Animals of SHMC with the following reference number: 20160225-115.

Note that full information on the approval of the study protocol must also be provided in the manuscript.

Flow Cytometry

Plots

Confirm that:

☒ The axis labels state the marker and fluorochrome used (e.g. CD4-FITC).

☒ The axis scales are clearly visible. Include numbers along axes only for bottom left plot of group (a 'group' is an analysis of identical markers).

☒ All plots are contour plots with outliers or pseudocolor plots.

☒ A numerical value for number of cells or percentage (with statistics) is provided.

Methodology

Sample preparation	Splenocytes isolated from vaccine immunized CMS5-VP1 tumor-bearing mice were stimulated with VP1, or PMA/Ionomycin as the positive control. Stimulated cells were surfaced stained with FVD-eFluor780, anti-CD3-eFluor506, anti-CD4-FITC , anti-CD8-PE. After surface staining, the cells were intracellular stained with anti-IL-2-Percp/Cy5.5 , anti-TNF-α-APC, anti-IFN-γ-BV421. Cells were washed twice and then resuspended with 200µl FBS.<br>For Treg staining, lymphocytes from lymph node were surface stained with eFluor780 conjugated FVD, anti-CD3-eFluor506, anti-CD4-FITC, and anti-CD8-PE, then stained with anti-FOXP3-BV421 and anti-LAP-PE/Cy7 . Cells were then resuspended 200µl PBS.
Instrument	Canto II flow cytometer(BD,#338962)
Software	FlowJo and Excel
Cell population abundance	N/A
Gating strategy	FSC/SSC for single lymphocytes and eFlour780-FVD negative cells were gating as lived cells, then T cells were gating as anti-CD3 positive, CD4+ T cells and CD8+ T cells were gated with anti-CD4-FITC and anti-CD8-PE antibodies. Then, CD4 or CD8 positive cells were graphed for IL-2, TNF-α and IFN-γ expression. For Tregs, lymphocytes were separated from inguinal lymph nodes near left hind limps, then live, CD3+CD4+ FoxP3+ T cells were gated and graphed for TGF-β expression with the marker LAP (surface latency-associated peptide of TGF-β)

☒ Tick this box to confirm that a figure exemplifying the gating strategy is provided in the Supplementary Information.
